# 67/m mit Bewusstseinstrübung bei Bronchialkarzinom: Vorbereitung auf die Zusatz-Weiterbildung Internistische Intensivmedizin: Fall 20

**DOI:** 10.1007/s00063-025-01393-3

**Published:** 2026-01-23

**Authors:** Fabian Perschinka, Michael Joannidis

**Affiliations:** Department Innere Medizin I, Division für Internistische Intensiv- und Notfallmedizin, Medizinische Universität Innsbruck, Anichstr. 35, 6020 Innsbruck, Österreich

**Keywords:** Hypoosmolare Hyponatriämie, Diagnostik, Therapie, Syndrom der inadäquaten Sekretion von antidiuretischem Hormon (SIADH), Osmotisches Demyelinisierungssyndrom

## Prüfungssimulation

### Fallschilderung

Ein 67-jähriger Mann wird auf die Intensivstation aufgenommen, nachdem er zu Hause somnolent aufgefunden wurde. In der Vorgeschichte findet sich ein bekanntes kleinzelliges Bronchialkarzinom mit Metastasenbildung, das aktuell mit Chemotherapie behandelt wird. Der Patient hat in den Tagen vor der Aufnahme auf der Intensivstation über zunehmende Müdigkeit, Übelkeit und Schwäche geklagt. Bei der klinischen Untersuchung ist er somnolent, aber erweckbar und zeigt eine leichte Verwirrtheit. Es gibt keine Hinweise auf fokale neurologische Ausfälle. In einer durchgeführten Computertomographie (CT) des Schädels werden keine Pathologien diagnostiziert. Der Volumenstatus des Patienten wird als „euvoläm“ eingeschätzt. Es liegt ein normofrequenter Sinusrhythmus bei einem Blutdruck von 128/73 mmHg vor.

Laborbefunde bei Aufnahme:

- Natrium 114 mmol/l (Referenzbereich [RB] 135–145 mmol/l)
- Kalium 3,0 mmol/l (RB 3,6–5,0 mmol/l)
- Kreatinin 1,2 mg/dl (RB 0,81–1,44 mg/dl)
- Harnstoff 20 mg/dl (RB 10–55 mg/dl)
- Glukose 90 mg/dl (RB nüchtern < 100 mg/dl)
- Serumosmolalität 260 mosm/kg (RB 275–295 mosm/kg)
- Urinosmolalität 520 mosm/kg (RB 50–1200 mosm/kg)
- Urinnatrium 45 mmol/l
- Thyreoideastimulierendes Hormon (TSH) 1,8 mIU/l (RB 0,4–4,0 mIU/l)
- Kortisol (morgens) 15 µg/dl (RB 4,8–19,5 µg/dl)

## Prüfungsfragen

- Wie lautet Ihre Verdachtsdiagnose? Begründen Sie Ihre Antwort
- Welche Therapieoptionen stehen zur Verfügung, um die akute hypotone Hyponatriämie zu behandeln? Unterscheiden Sie in Bezug auf die zugrunde liegenden Krankheitsbilder
- Beschreiben Sie die Therapie der akuten hypotonen Hyponatriämie mit schwerer Symptomatik. Wie unterscheidet sich davon die Therapie bei hypovolämen Patient*innen?
- Welche potenziellen Gefahren bestehen bei der Korrektur einer profunden Hyponatriämie? Wie sollten Sie die Natriumspiegel sicher anheben und ggf. auf eine Überkorrektur reagieren?
- Wie können Sie den Verlauf der Therapie überwachen, um Komplikationen zu vermeiden?
- Was sind Ihre Langzeitüberlegungen in Bezug auf eine Therapie für Patient*innen mit Syndrom der inadäquaten Sekretion von antidiuretischem Hormon, und wie kann eine erneute Hyponatriämie in Zukunft verhindert werden?

### Antworten

#### Wie lautet Ihre Verdachtsdiagnose? Begründen Sie Ihre Antwort

**Abb. 1:**
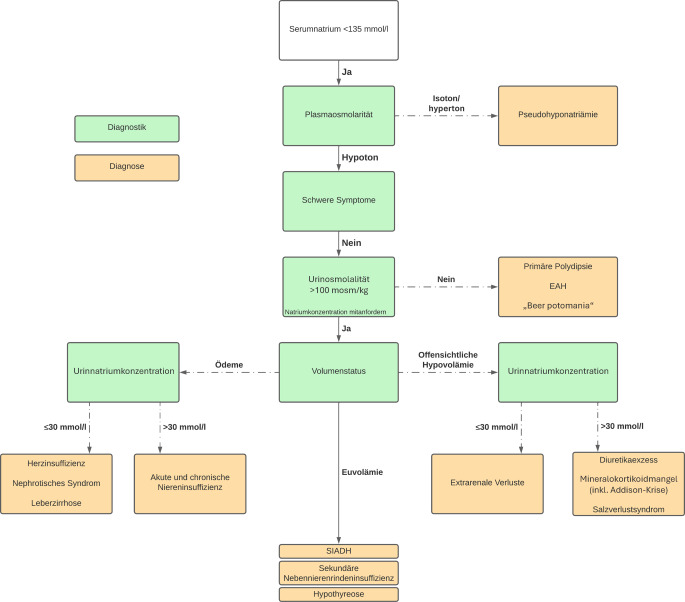
Diagnostisches Fließdiagramm. *EAH* „exercise-associated hyponatremia“, *SIADH* Syndrom der inadäquaten Sekretion von antidiuretischem Hormon

- In der Zusammenschau von Laborbefunden und Anamnese ergibt sich die **Verdachtsdiagnose eines Syndroms der inadäquaten Sekretion von antidiuretischem Hormon (SIADH) als Ursache der Hyponatriämie** (s. Abb. 1):
  - Die Verdachtsdiagnose eines SIADH basiert auf dem **Vorliegen einer hypotonen Hyponatriämie und einer Urinosmolalität** **>** **100** **mosm/kg bei gleichzeitig bestehender Euvolämie und hoher Natriumausscheidung im Harn.**
  - Die bei einer Urinosmolalität > 100 mosm/kg und euvolämem Volumenstatus in Betracht zu ziehenden Differenzialdiagnosen einer **Nebennierenrindeninsuffizienz** oder **Hypothyreose** erscheinen **angesichts des normwertigen TSH und Kortisols** als Ursache der Hyponatriämie unwahrscheinlich.
  - Eine **primäre Polydipsie oder eine „beer potomania“** scheiden als Differenzialdiagnosen aufgrund der **Urinosmolalität** **>** **100** **mosm/kg** aus.
- Beim SIADH im Rahmen eines **kleinzelligen Bronchialkarzinoms** kommt es zu einer **paraneoplastischen Ausschüttung von antidiuretischem Hormon** (ADH) durch die Tumorzellen.
- Dies führt zu einer **verstärkten Rückresorption von freiem Wasser in den Nieren**, wodurch das Natrium im Serum verdünnt wird. Trotz eines normovolämen Flüssigkeitsstatus entsteht so eine **hypotone Hyponatriämie**. Die physiologische Hemmung der ADH-Sekretion durch eine niedrige Plasmaosmolalität bleibt aus, da die ADH-Produktion nicht hypothalamisch, sondern ektopisch erfolgt [1, 2].

**Therapie:**

- Die primäre Behandlung besteht in der Therapie der Grunderkrankung.
- Ist dies nicht möglich oder kommt es trotz Behandlung zu einer Persistenz, kann das SIADH mit Flüssigkeitsrestriktion, oraler NaCl-Aufnahme oder oraler Einnahme von Harnstoff behandelt werden [1].
- Eine weitere Therapie-Alternative ist der Einsatz von Vaptanen [1].
- Bei fortschreitender Grunderkrankung sollte die Therapie stets unter Berücksichtigung der verbleibenden Lebensqualität individuell angepasst und das weitere Vorgehen in enger Abstimmung mit dem/der Patienten/Patientin gemeinsam festgelegt werden. Ein aggressives Elektrolytmanagement ist hierbei nicht angeraten.

#### Welche Therapieoptionen stehen zur Verfügung, um die akute hypotone Hyponatriämie zu behandeln? Unterscheiden Sie in Bezug auf die zugrunde liegenden Krankheitsbilder

**Therapieentscheidung je nach Symptomatik:**

- Mittelschwere bis schwere Symptomatik:
  - **Sofortige Therapie** mit **3** **%iger NaCl-Lösung** erforderlich
- Leichte oder fehlende Symptome:
  - Zunächst **weiterführende Diagnostik** vor Therapieeinleitung
  - Therapieentscheidung abhängig vom **zugrunde liegenden Krankheitsbild**

**Therapie nach zugrunde liegendem Krankheitsbild:**

SIADH:

- Sofern möglich, steht die **Behebung der Ursache der ADH-Sekretion** im Vordergrund
- **Früher Beginn** einer **Flüssigkeitsrestriktion**, parallel zur Ursachensuche
- Bei fehlender Normalisierung des Serumnatriums:
  - **Harnstoffgabe**: 0,25–0,50 g/kgKG täglich
    oder
  - **Schleifendiuretika** **+** **orales NaCl**
- Bei ausbleibendem Therapieerfolg:
  - Einsatz eines **Vasopressinrezeptorantagonisten** (z. B. Tolvaptan)
  - Achtung: **Startdosis 7,5** **mg statt 15** **mg** Tolvaptan, um **raschen Natriumanstieg zu vermeiden** [3, 4]

Zerebrales Salzverlustsyndrom:

- **Ausgleich des extrazellulären Flüssigkeitsverlusts** und der **Hypovolämie**
- Wahl der Infusionslösung **je nach Urinosmolalität**:
  - < 300 mosm/kg: **NaCl 0,9** **%** oder **balancierte kristalloide Lösung**
  - ≥ 300 mosm/kg: **hypertone Kochsalzlösung**
- Ziel: Osmolalität der Infusion > Urinosmolalität

Intravasale Hypovolämie:

- **Volumenersatztherapie mit 0,5–1** **ml/kgKG pro h NaCl 0,9** **%** [5]
- Ziel: **Reduktion der ADH-Sekretion**
- Bei zugrunde liegender Erkrankung (z. B. **Leberzirrhose**):
  - **Behandlung der Grunderkrankung notwendig**

Übermäßige Flüssigkeitszufuhr:

- Ursache: Flüssigkeitszufuhr übersteigt Ausscheidung → **Verdünnung des Serumnatriums**
- Therapie:
  - **Flüssigkeitsrestriktion** [5] und **orale Kochsalzzufuhr**
- Bei „**tea and toast syndrome**“ oder „**beer potomania**“:
  - Zusätzlich **Ernährungsberatung** erforderlich

Belastungsassoziierte Hyponatriämie:

- **Meist mild und selbstlimitierend**
- Nach Ausschluss einer Hypovolämie:
  - Bei leichter bis moderater Ausprägung: **Flüssigkeitsrestriktion** [6, 7]

Die therapeutischen Maßnahmen in Abhängigkeit von der Ätiologie sind in Tab. 1 zusammengefasst.

**Tab. 1:** Therapie der Hyponatriämie in Abhängigkeit von der Ätiologie

Ätiologie	Volumenstatus	Therapie
Syndrom der inadäquaten Sekretion von antidiuretischem Hormon (SIADH)	Euvolämie	Flüssigkeitsrestriktion, ggf. Harnstoff oder Schleifendiuretika + orales NaCl, Vaptane
Zerebrales Salzverlustsyndrom	Hypovolämie	Volumensubstitution mit NaCl
Diuretikaexzess		
Mineralokortikoidmangel (inkl. Addison-Krise)		
Polydipsie	Normo‑/Hypervolämie	Flüssigkeitsrestriktion, psychologische/edukative Maßnahmen (Ernährungsberatung)
Belastung	Variabel	Abwarten, Flüssigkeitsrestriktion
Herzinsuffizienz	Ödeme bei vermindertem intravasal zirkulierendem Volumen	Behandlung der Grunderkrankung
Nephrotisches Syndrom		
Leberzirrhose		
Hypothyreose	Euvolämie	Behandlung der Grunderkrankung
Sekundäre Nebennierenrindeninsuffizienz		

#### Beschreiben Sie die Therapie der akuten hypotonen Hyponatriämie mit schwerer Symptomatik. Wie unterscheidet sich davon die Therapie bei hypovolämen Patient*innen?

- Die Therapie der akuten hypotonen Hyponatriämie wird **anhand der Schwere und des zeitlichen Auftretens der Symptome sowie des Volumenstatuses** gewählt.
- Bei mittelschweren und schweren Symptomen steht die **zügige Therapieinitiierung** im Vordergrund.
  - Bei **Hyper- und Euvolämie** können 150 ml **NaCl 3** **%** über 20 min ohne Bedenken verabreicht werden. Eine Gabe von 150 ml NaCl 3 % kann bis zu 4‑mal wiederholt werden, wobei ein Abstand von 20 min zwischen den Infusionen liegen sollte. Es wird eine Messung des Serumnatriums nach jeder Gabe von NaCl 3 % empfohlen [5].
  - Bei Personen mit Über- oder Untergewicht kann auch eine **gewichtsadaptierte Verabreichung** von 2 ml/kgKG anstatt der 150 ml pro Infusion erfolgen [8]. Als Faustregel kann angenommen werden, dass 1 ml/kgKG NaCl 3 % die Serumnatriumkonzentration etwa um 1 mmol/l anhebt.
  - In der **klinisch manifesten Hypovolämie** wird der Volumenmangel zuerst mit **isotonen Kristalloiden**, bevorzugt NaCl 0,9 % (0,5–1 ml/kgKG pro h), behandelt, um den Volumenstatus zu korrigieren und die hypovolämiebedingte ADH-Ausschüttung zu verringern.
- Kommt es nach erfolgter Therapie und Normalisierung des Serumnatriums **zu keiner Besserung der neurologischen Symptome**, sollte unbedingt eine **weitere Bildgebung** (Magnetresonanztomographie [MRT]/CT) durchgeführt werden, um Differenzialdiagnosen für die persistierende Bewusstseinsstörung auszuschließen (z. B. osmotische Myelinolyse, zerebrale Metastasen).

##### Merke.

Bei mittelschweren oder schweren Symptomen steht die rasche Therapieinitiierung zur Anhebung der Serumosmolarität unabhängig von der Ätiologie im Vordergrund.

##### Cave.

Als Folge der Flüssigkeitsgabe bei hypovolämen Patient*innen kann die ADH-bedingte Antidiurese sistieren. Mit der wiedereinsetzenden Diurese (> 100 ml/h) geht ein schnellerer Anstieg der Serumnatriumkonzentration einher. In diesem Fall sind Natriumkontrollen alle 2 h zu empfehlen und ggf. Maßnahmen bei Überkorrektur zu ergreifen [9].

#### Welche potenziellen Gefahren bestehen bei der Korrektur einer profunden Hyponatriämie? Wie sollten Sie die Natriumspiegel sicher anheben und ggf. auf eine Überkorrektur reagieren?

**Gefahr: osmotisches Demyelinisierungssyndrom (ODS)**

Ursache:

- Bei chronischer hypotoner Hyponatriämie passen sich die Zellen an die niedrige Osmolalität an → Eine **zu schnelle Korrektur** überfordert die Readaptation und die Zellen schrumpfen → **Zellschädigung**.
- **Klinische Manifestationen** [10–12]:
  - Muskelschwäche (insbesondere der Extremitäten)
  - Dysarthrie, Dysphagie
  - Im Extremfall: **Atemstillstand**

Sichere Korrekturgrenzen [5]:

- Max. **+** **10** **mmol/l in den ersten 24** **h**
- Max. **+** **8** **mmol/l in den darauffolgenden 24** **h** (bis ein Serumnatrium von 130 mmol/l erreicht ist)

Maßnahmen bei Überkorrektur:

- **Sofortiger Stopp** der NaCl-Therapie
- Reversion der Überkorrektur:
  - Orale Wasserzufuhr oder
  - **Glukose 5** **% i.v.** (10 ml/kgKG) über 1 h
- Bei unzureichender Wirkung:
  - **Desmopressin** 2 µg alle 8 h in Erwägung ziehen

##### Cave.

Als Grenze ist ein maximaler Anstieg um 10 mmol/l innerhalb der ersten 24 h und um 8 mmol/l in den darauffolgenden 24 h (bis eine Serum-Natriumkonzentration von 130 mmol/l erreicht wird) zu beachten. Um die Grenzen einzuhalten, empfiehlt sich eine Messung nach jeder Infusion!

#### Wie können Sie den Verlauf der Therapie überwachen, um Komplikationen zu vermeiden?

Die Menge der zu verabreichenden Infusionslösung kann auf verschiedene Arten abgeschätzt werden.

Vereinfacht kann angenommen werden, dass 1 ml/kgKG NaCl 3 % die Serumnatriumkonzentration um etwa 1 mmol/l anhebt.

Zur präzisieren Abschätzung wird die Zuhilfenahme der Adrogué-Madias-Formel [13] empfohlen.

Beispiel eines 67-jährigen männlichen Patienten mit Körpergewicht von 80 kg:

$$\Delta \,\textit{Serum}\,\left(Na^{+}\right)=\frac{513\,mmol\left(Na^{+}\right)-114\,mmol\left(Na^{+}\right)}{48+1}$$

$$\frac{\left(10\,mmol\,\textit{gew{\"u}nschtes}\,\Delta Na^{+}\right)}{\left(8{,}14\,\textit{errechnetes}\,\Delta \,\textit{Serum}\,Na^{+}\right)}=\left(1{,}23l\,\textit{der}\,\textit{Infusionsl{\"o}sung}\right)$$

**Bei Besserung der Symptome:**

- **Beendigung** der Therapie mit NaCl 3 %
- **Serumnatriumkontrollen:**
  - **Alle 6** **h bis zu einer Serumnatriumkonzentration** **>** **125** **mmol/l**
  - Anschließend **täglich**, sobald die Serumnatriumkonzentration stabil ist [5]

**Bei ausbleibender Besserung:**

- **Fortführung der Therapie mit NaCl 3** **%** bis:
  - Serumnatrium ≥ 130 mmol/l
  - Steigerung um + 10 mmol/l in 24 h
  - Klinische Besserung der Symptome
- Empfohlen:
  - → **Serumnatriumkontrolle nach jeder Infusion und alle 4** **h**

Wenn weiterhin keine Besserung:

- Durchführung einer **bildgebenden Diagnostik** (z. B. kraniale CT/MRT)

**Zusätzliche Verlaufskontrollen:**

- **Quantitative Messung der Harnausscheidung**:
  - Frühzeitige Erkennung einer **Polyurie**
  - Vermeidung eines **zu raschen Serumnatriumanstiegs**

##### Merke.

Als Faustregel kann angenommen werden, dass 1 ml/kgKG NaCl 3 % die Serumnatriumkonzentration etwa um 1 mmol/l anhebt.

#### Was sind Ihre Langzeitüberlegungen in Bezug auf eine Therapie für Patient*innen mit Syndrom der inadäquaten Sekretion von antidiuretischem Hormon, und wie kann eine erneute Hyponatriämie in Zukunft verhindert werden?

**Therapiekaskade bei persistierendem SIADH** [5, 14]**:**

- Schritt 1: **Flüssigkeitsrestriktion**
  - < 800 ml/Tag
  - **Nicht geeignet bei kürzlicher subarachnoidaler Blutung**
- Schritt 2: **Salzsubstitution**
  - Zusätzlich zur Nahrung: **3** **g Salz zu den Hauptmahlzeiten**
- Schritt 3: **Schleifendiuretika**
  - Ziel: **Reduktion der Urinosmolalität**
  - Beispiel: **Furosemid 20** **mg 2‑mal pro Tag**
  - Beachte: Wirkung von Salzaufnahme und Flüssigkeitsrestriktion kann dadurch reduziert werden
- Schritt 4: **Harnstoff**
  - 15–30 g/Tag
  - Ziel: **Steigerung des Harnvolumens** zur Natriumanhebung
- Schritt 5: **Vaptane** (z. B. Tolvaptan 7,5 mg):
  - Langzeitanwendung nicht empfohlen
  - Nur in Erwägung ziehen bei
    - **Serumnatrium <** **120** **mmol/l** trotz anderer Maßnahmen oder
    - **persistierenden neurologischen Symptomen**
  - In palliativen Situationen:
    - → **Monitoringintervall individuell festlegen**, z. B. **alle 3 Tage**, unter Berücksichtigung der **Lebensqualität**

Eine Auflistung der Maßnahmen und der dazugehörigen Begründungen findet sich in Tab. 2.

**Tab. 2:** Therapieeskalation bei Syndrom der inadäquaten Sekretion von antidiuretischem Hormon

Schritt	Maßnahme	Begründung
1	Flüssigkeitsrestriktion < 800 ml/Tag	Basismaßnahme, um eine Verdünnung des Natriums zu verhindern
2	NaCl-Gabe (oral, z. B. 3‑mal 3 g)	Steigerung der Serumnatriumwerte durch gesteigerte Einnahme
3	+ Schleifendiuretikum (Furosemid)	Osmolalität im Harn senken
4	Harnstoff (15–30 g/Tag)	Steigerung des Harnvolumens
5	Vaptan (z. B. Tolvaptan 7,5 mg)	Wirkung der gesteigerten Sekretion von antidiuretischem Hormon aufheben – nur bei Versagen, da „off label use“ in Langzeittherapie

## References

[1] Adrogué HJ, Madias NE (2023) The Syndrome of Inappropriate Antidiuresis. N Engl J Med 389(16):1499–1509 37851876 10.1056/NEJMcp2210411

[2] Grohé C, Berardi R, Burst V (2015) Hyponatraemia—SIADH in lung cancer diagnostic and treatment algorithms. Crit Rev Oncol Hematol 96(1):1–8 26070626 10.1016/j.critrevonc.2015.04.005

[3] Hanna RM, Velez JC, Rastogi A, Nguyen MK, Kamgar MK, Moe K et al (2020) Equivalent efficacy and decreased rate of overcorrection in patients with syndrome of inappropriate secretion of antidiuretic hormone given very low-dose tolvaptan. Kidney Med 2(1):20–28 32734225 10.1016/j.xkme.2019.09.004 PMC7380356

[4] Sterns RH (2018) Tolvaptan for the syndrome of inappropriate secretion of antidiuretic hormone: is the dose too high? Am J Kidney Dis 71(6):763–765 29801549 10.1053/j.ajkd.2018.02.355

[5] Spasovski G, Vanholder R, Allolio B, Annane D, Ball S, Bichet D et al (2014) Clinical practice guideline on diagnosis and treatment of hyponatraemia. Nephrol Dial Transplant 29(2):i1–i39 24569496 10.1093/ndt/gfu040

[6] Bennett BL, Hew-Butler T, Rosner MH, Myers T, Lipman GS (2020) Wilderness Medical Society Clinical Practice Guidelines for the Management of Exercise-Associated Hyponatremia: 2019 Update. Wilderness Environ Med 31(1):50–62 32044213 10.1016/j.wem.2019.11.003

[7] Hew-Butler T, Rosner MH, Fowkes-Godek S, Dugas JP, Hoffman MD, Lewis DP et al (2015) Statement of the Third International Exercise-Associated Hyponatremia Consensus Development Conference, Carlsbad, California, 2015. Clin J Sport Med 25(4):303–320 26102445 10.1097/JSM.0000000000000221

[8] Pelouto A, Refardt JC, Christ-Crain M, Zandbergen AAM, Hoorn EJ (2023) Overcorrection and undercorrection with fixed dosing of bolus hypertonic saline for symptomatic hyponatremia. Eur J Endocrinol 188(3):322–330 36881992 10.1093/ejendo/lvad028

[9] Perschinka F, Köglberger P, Klein SJ, Joannidis M (2023) Hyponatremia : Etiology, diagnosis and acute therapy. Med Klin Intensivmed Notfmed 118(6):505–517 37646802 10.1007/s00063-023-01049-0 PMC10501960

[10] George JC, Zafar W, Bucaloiu ID, Chang AR (2018) Risk factors and outcomes of rapid correction of severe hyponatremia. Clin J Am Soc Nephrol 13(7):984–992 29871886 10.2215/CJN.13061117 PMC6032596

[11] Alleman AM (2014) Osmotic demyelination syndrome: central pontine myelinolysis and extrapontine myelinolysis. Semin Ultrasound CT MR 35(2):153–159 24745890 10.1053/j.sult.2013.09.009

[12] Popescu BF, Bunyan RF, Guo Y, Parisi JE, Lennon VA, Lucchinetti CF (2013) Evidence of aquaporin involvement in human central pontine myelinolysis. Acta neuropathol commun 1:40 24252214 10.1186/2051-5960-1-40 PMC3893459

[13] Adrogué HJ, Madias NE (2000) Hyponatremia. N Engl J Med 342(21):1581–1589 10824078 10.1056/NEJM200005253422107

[14] Krisanapan P, Vongsanim S, Pin-On P, Ruengorn C, Noppakun K (2020) Efficacy of Furosemide, Oral Sodium Chloride, and Fluid Restriction for Treatment of Syndrome of Inappropriate Antidiuresis (SIAD): An Open-label Randomized Controlled Study (The EFFUSE-FLUID Trial). Am J Kidney Dis 76(2):203–212 32199708 10.1053/j.ajkd.2019.11.012

